# Supplementary material for: Aminoglycosides in the Intensive Care Unit: What Is New in Population PK Modeling?
Source: Antibiotics (Basel). 2021 Apr 29;10(5):507. doi: 10.3390/antibiotics10050507 (PMC8145041; doi:10.3390/antibiotics10050507)
Supplement: Supplementary file 1 [file antibiotics-10-00507-s001.zip › antibiotics-1203031-supplementary.pdf]

Supplementary Information:

Table S1 : Checklist of information to be included when reporting a clinical pharmacokinetic study based on *ClinPK* [13]

Drug	Study	Title/Abstract		Background			Methods										Results						Discussion/Conclusion		Other Information
		1	2	3	4	5	6	7	8	9	10	11	12	13	14	15	16	17	18	19	20	21	22	23	24
Amikacin	Boidin C [16]	●	●	●	●	●	●	○	●	○	●	●	●	●	●	●	○	○	●	●	NA	NA	●	●	○
	Roger C [17]	●	●	●	●	●	●	○	●	●	●	●	●	○	●	●	○	○	●	●	●	NA	●	●	●
	Carrié C [18]	●	●	●	●	●	●	○	●	●	●	●	●	●	●	●	○	○	●	●	●	NA	●	●	●
	Aréchiga-Alvarado NA [19]	●	●	●	●	●	●	○	●	●	●	●	●	●	●	●	○	○	●	●	NA	NA	●	●	●
	Petitcollin A [20]	●	●	●	●	●	●	○	●	●	●	●	●	●	●	●	○	○	●	●	NA	NA	●	●	●
	French MA [21]	●	●	●	●	●	●	●	○	○	○	●	○	○	●	●	○	○	●	●	NA	NA	●	●	●
Gentamicin	Hodiamont CJ [22]	●	●	●	●	●	●	○	●	●	●	●	●	●	●	●	○	○	●	●	●	NA	●	●	●
	Teigen MM [23]	●	●	●	●	●	●	○	●	●	●	●	●	●	●	●	○	○	●	●	●	NA	●	●	●
	Rea RS [24]	●	●	●	●	●	●	○	●	●	●	●	●	●	●	●	○	○	●	●	NA	NA	●	●	●
	Bos JC [25]	●	●	●	●	●	●	○	●	●	●	●	●	●	●	●	○	○	●	●	NA	NA	●	●	●
	Hodiamont CJ [26]	●	●	●	●	●	●	○	●	●	●	●	●	●	●	●	○	○	●	●	NA	NA	●	●	●
	Roberts JA [27]	●	●	●	●	●	●	○	●	●	●	●	●	●	●	●	○	○	●	●	●	NA	●	●	●
	Barletta JF [28]	●	●	●	●	●	●	○	○	●	○	●	●	●	●	●	○	○	●	●	NA	NA	○	●	○
	Gomes A [29]	●	●	●	●	●	●	○	●	●	●	●	●	●	●	●	○	○	●	●	NA	NA	●	●	●
	Watling SM [30]	●	●	●	●	●	●	○	●	●	●	●	●	●	●	●	○	○	●	●	NA	NA	●	●	○
	Kisor DF [31]	●	●	●	●	●	●	○	○	●	●	●	○	●	●	●	○	○	●	●	NA	NA	●	●	○
	French MA [21]	●	●	●	●	●	●	●	○	○	○	●	○	○	●	●	○	○	●	●	NA	NA	●	●	●
Tobramycin	Conil JM [32]	●	●	●	●	●	●	○	●	●	●	●	●	●	●	●	○	○	●	●	NA	NA	●	●	●
	Aarons L [33]	●	●	●	●	●	●	○	○	●	●	●	●	●	●	●	○	○	●	●	NA	NA	●	●	●
	Hennig S [34]	●	●	●	●	●	●	○	●	●	●	●	●	●	●	●	○	○	●	●	NA	NA	●	●	●

● Information included
○ Information not included or not found
NA: Not Applicable

Table S2: Characteristics of the population pharmacokinetic models developed by the studies included in this review (one compartment)

Drug	Study	CL (L/h)			V_d (L)			K_{el}			IIV		RV		
		Formula	Parameter	Value	Formula	Parameter	Value	Formula	Parameter	Value	CL (%)	V_d (%)	Exponential	Proportional	Additive
Amikacin	Aréchiga-Alvarado NA [19]	$CL = \theta_1 X (CL_{CR}/130)^{\theta_3}$	θ_1	7.1	$V_d = \theta_2 X (IBW/68)^{\theta_4}$	θ_2	20.3	NR	NR	NR	27.2	33.6	–	–	1.78 mg/L
			θ_3	0.84		θ_4	2.94								
Gentamicin	Teigen MM [23]	$CL_{NHD} = \theta_1 X (CL_{CR}/0.53)$	θ_1	0.453	$V_d = \theta_3$	θ_3	23.5	NR	NR	NR	$\omega^2 = 0.264$	$\omega^2 = 0.0256$	–	0.0804[a]	0.00659[a]
		$CL_{HD} = \theta_2$	θ_2	4.69											
	Rea RS [24]	$CL = (\theta_{1\_CLMAX} X GFR^{1.2})/(\theta_{2\_EGFRHILL50} + GFR^{1.2})$	θ_{1_CLMAX}	3.14	$V_d = \theta_3 X (BW/70)$	θ_3	53	NR	NR	NR	83.7	64.4	–	24.3	0.381 µg/mL
			$\theta_{2\_EGFRHILL50}$	54.8											
	Bos JC [25]	$CL = \theta_1 X (1 + \theta_{2\_Factor\ associating\ Gentamicin\ CL\ and\ CLcr} X (CL_{CR} - 74)$	θ_1	5.7	NR	V_d	19	NR	NR	NR	74	49	–	32	0.056 mg/L
			$\theta_{2\_Factor\ associating\ Gentamicin\ CL\ and\ CLcr}$	0.0091											
	Barletta JF [28]	NR	CL	5.41	NR	V_d	Gentamicine:34.3<br>Tobramycine : 17.3	NR	NR	NR	29.3	11.9	–	–	144%
	Gomes A [29]	NR	CL_{fr} [b]	0.698	NR	V_d	0.312 L/kg LBMc	NR	NR	NR	NR	NR	NR	NR	NR
	Watling SM [30]	NR	NR	NR	NR	V_d	0.34 L/Kg	$K_{el} = \theta_{KS} X CL_{CR} + \theta_{Ki}$	θ_{KS}	0.00218	NR	NR	NR	NR	NR
								NR	θ_{Ki}	0.007					
	Kisor DF [31]	NR	CL	3.01	NR	V_d	0.376 L/kg	NR	K_{el}	0.203	NR	NR	NR	NR	NR

BW Body Weight, *CL* Clearance, *CLcr* Creatinine Clearance (mL/min/m2), *CL_{HD}* Hemodialysis Clearance, *CL_{MAX} Maximum Clearance*, *CL_{NHD}* Non hemodialysis Clearance, *EGFRHILL50* GFR yielding half of CLMAX (mL/min), *GFR* Glomerular Filtration rate (mL/min) *IBW* Ideal Body Weight, *IIV* Interindividual variation, *K_{el}* Elimination Constant, *K_S* K Slope, *K_i* Intercept, *LBMc* Lean body mass according to the equation of Chennavasin corrected for fat distribution, *NR* Not Reported, *RV* Residual Variability, Vd Volume of Distribution

[a] Variance

[b] renal gentamicin clearance as a fraction of creatinine clearance

[c] PK Model was constructed with samples from Gentamicin and Tobramycin

Table S3: Characteristics of the population pharmacokinetic models developed by the studies included in this review (two-compartment)

Drug	Study	CL (L/h)			V_1 (L)			V_2 (L)			K_{el}		
		Formula	Parameter	Value	Formula	Parameter	Value	Formula	Parameter	Value	Formula	Parameter	Value
Amikacin	Boidin C [16]	$CL = \theta_1 X (CL_{CG}/73.6)^{\theta_2}$	θ_1	2.6	$V_1 = \theta_3 X (BSA/1.93)$	θ_3	23	$V_1 = V_2$	V_2	23	NR	NR	NR
			θ_2	0.85									
	Roger C [17]	$CL = \theta_{CLHF} X [(TBW/80)^{0.75}] + \theta_{CLHDF} X [(TBW/75)^{0.75}]$	θ_{CLHF}	4.69	NR	V_1 (central)	25.2	NR	NR	NR	NR	NR	NR
			θ_{CLHDF}	4.45									
	Carrié C [18]	$CL= \theta_{CLpop} X (CL_{CR}/66.7)^{0.69}$	θCLpop	NR	$V_1= \theta_{V1pop} X (CL_{CR}/66.7)^{0.22} X (ABW/79.6)^{0.48}$	θ_{V1pop}	NR	NR	$V_{n\text{-}crrt}$	11.7	NR	NR	NR
			$CL_{n\text{-}crrt}$	3.02	NR	$V_{n\text{-}crrt}$	23.2	NR	V_{crrt}	14.1			
			CL_{crrt}	2.32	NR	V_{crrt}	25.5						
	Petitcollin A [20]	NR	CL	4.98	NR	Vc	10.2	NR	V_2	14.98	NR	K_{10}	0.488
	French MA [21]	NR	TBCL	33.45[d]	NR	Vc	0.305 L/Kg	NR	NR	NR	NR	K_{10}	0.1
Gentamicin	Hodiamont CJ [22]	$CL= \theta_{noCVVH} X (IBW/70)^{0.75}$	θ_{noCVVH}	1.15	$V_1 = \theta_1 X (IBW/70) X (ALBM/22)^{-0.833}$	θ_1	21.2 L/70 Kg	NR	V_2	18.4 L/70 kg	NR	NR	NR
		$CL= \theta_{CVVH} X (IBW/70)^{0.75}$	θ_{CVVH}	2.13									
	Hodiamont CJ [26]	NR	CL	2.3	NR	V_1	21.6	NR	V_2	10.2	NR	NR	NR
	Roberts JA [27]	$TVCL_{NEDD\text{-}f} = \theta_1 X (LBW/55) X FCR$	$CL_{EDD\text{-}F}$	2.59	NR	Vc	14.1	NR	Vp	32.8	NR	NR	NR
		$TVCL = TVCL_{NEDD\text{-}f} + TVCL_{EDD\text{-}f}$	$CL_{NEDD\text{-}F}$	0.24									
			TVCL	2.83									
	French MA [21]	NR	TBCL	29.53 [d]	NR	Vc	0.257 L/Kg	NR	NR	NR	NR	K_{10}	0.18
Tobramycin	Conil JM [32]	$TVCL = \theta_1 + (\theta_2 X (COCK\text{-}94)) + (\theta_3 X (HEIG\text{-}172))$	θ_1	3.83	$TVV_1 = \theta_4$	θ_4	25.5	NR	NR	NR	NR	NR	NR
			θ_2	0.02									
			θ_3	0.052									
	Aarons L [33]	CL = θ1 X CLCR	θ_1	0.059 [e]	NR	V_1	0.327	NR	NR	NR	NR	NR	NR
	Hennig S [34]	$CL\_f/m = \theta_{CL\_f/m} x (FFM/70)^{0.952} x [1 + \theta_{AGE} x (AGE - 18)] x (SCRmean/SCR)^{0.222}$	θ_{CL_f}	8.1 L/h/70 Kg	$V_1 (f/m) = \theta_{V1,f/m} x (FFM/70)$	θ_{V1_f}	20.2 L/70 Kg	$V_2 = \theta_{V2} X (FFM/70)^{\theta FFM}$	θ_{V2}	10.0 L/70 Kg	NR	NR	NR
			θ_{CL_m}	9.5 L/h/70 Kg		θ_{V1_m}	25.2 L/70 Kg		θ_{FFM}	0.975			
			θ_{Age_u18}	-0.021									
			θ_{Age_o18}	-0.01									

ABW Adjusted Bodyweight, *ALBM* Albumine level, *Age_o18* Age over 18 years old, *Age_u18* Age under 18 years old, *BSA Body Surface Area*, CL_{CG} or CL_{CR} or *COCK* Creatinine clearance estimated by the original Cockcroft-Gault Equation, CL_{CRRT} Clearance of patients under CRRT (Continuous renal replacement therapy), CL_{CVVH} Clearance when patient is on CVVH (continuous venovenous haemofiltration), $CL_{EDD\text{-}f}$ Clearance when patient is on EDD-f (extended daily diafiltration) , *CLHDF* Clearance on hemodiafiltration, *CLHF* Clearance on hemofiltration, $CL_{NEDD\text{-}f}$ Clearance when patient is not on EDD-f, CL_{noCVVH} Clearance when patient is off CVVH, $CL_{n\text{-}CRRT}$ Clearance of patients not under CRRT, *CL_f* Clearance if patient is female, *CL_m* Clearance if patient is male, *FCR* Inverse of the final plasma creatine concentration recorded in μmol/L before commencement of EDD-f, *FFM* Fat-free mass, *HEIG* Height, *IBW* Ideal Bodyweight, *Q* Intercompartment clearance, *TBCL* Total Body Clearance, *TBW* Total Bodyweight, *V1_f* Apparent Volume of distribution of the central compartment if patient is female, *V1_m* Apparent Volume of distribution of the central compartment if patient is male, V_1 *or Vc* central volume, V_{CRRT} distribution volume of patients under CRRT, $Vn_{\text{-}CRRT}$ distribution volume of patients not under CRRT, *Vp* peripheral volume

[a] Of note, the author set the peripheral volume V_2 equal to V_1.

[b] Coefficient of variation, calculated as the square root of $(e^{\omega}-1)$*100%

[c] Variance of the residual error

[d] Estimated as ml/min

[e] Proportionality constant relating creatinine clearance (ml/min) to drug clearance (L/h)

Table S3: Characteristics of the population pharmacokinetic models developed by the studies included in this review (two-compartment) (continued)

Drug	Study	Q (L/h)			K_{12} (h^{-1})			K_{21} (h^{-1})			IIV				RV		
		Formula	Parameter	Value	Formula	Parameter	Value	Formula	Parameter	Value	CL (%)	V_1 (%)	V_2 (%)	Q (%)	Exponential (%)	Proportional (%)	Additive (mg/L)
Amikacin	Boidin C [16]	NR	θ_4	0.5	NR	NR	NR	NR	NR	NR	NR	NR	NR	NR	NR	NR	NR
	Roger C [17]				NR	K_{12} (cp)	0.89	NR	K_{21} (pc)	2.38	NR	NR	NR	NR	NR	NR	NR
	Carrié C [18]	NR	$Q_{n\text{-}crrt}$	1.05	NR	NR	NR	NR	NR	NR	$\omega^2_{n\text{-}crrt}$ = 0.345	$\omega^2_{n\text{-}crrt}$ = 0.047	$\omega^2_{n\text{-}crrt}$ = 0.695	$\omega^2_{n\text{-}crrt}$ = 0.813	–	N-CRRT = 0.132	–
			Q_{crrt}	1.19							ω^2_{crrt} = 0.221	ω^2_{crrt} = 0.081	ω^2_{crrt} = 0.380	ω^2_{crrt} = 0.418 L/h		CRRT = 0.232	
	Petitcollin A [20]	NR	Q	12.85	NR	K_{12}	1.26	NR	K_{21}	0.858	NR	NR	NR	NR	–	10.9	0.214
	French MA [21]				NR	K_{12}	0.016	NR	K_{21}	0.005	NR	NR	NR	NR	NR	NR	NR
Gentamicin	Hodiamont CJ [22]	NR	Q	1.96*	NR	NR	NR	NR	NR	NR	CL_{noCVVH} = 42.5 [b]<br>CL_{CVVH} = 29.5 [b]	17.2	NR<br>NR	NR<br>NR	–	33.8	–
	Hodiamont CJ [26]	NR	Q	1.3	NR	NR	NR	NR	NR	NR	75.0 [b]	27.0 [b]	NR	NR	–	19.4	0.13
	Roberts JA [27]	NR	Q	2.76	NR	NR	NR	NR	NR	NR	39.9 [b]	16.4 [b]	20.5 [b]	110.5 [b]	–	20.8	–
	French MA [21]	NR	NR	NR	NR	K_{12}	0.025	NR	K_{21}	0.01	NR	NR	NR	NR	NR	NR	NR
Tobramycin	Conil JM [32]	NR	NR	NR	NR	NR	NR	NR	NR	NR	ω^2=0.095	ω^2 = 0.045	Fixed	Fixed	–	0.056 [c]	–
	Aarons L [33]	NR	NR	NR	NR	K_{12}	0.012	NR	K_{21}	0.027	32	3	NR		–	21	–
	Hennig S [34]	$Q = \theta_Q$ X $(FFM/70)^{\theta FFM}$	θ_Q	1.5*	NR	NR	NR	NR	NR	NR	CL_m = NR	V_{1_m} = NR	58.5	41.8	–	20.4	–
			θ_{FFM}	0.975							CL_f = 25.9	V_{1_f} = 15.2					

ABW Adjusted Bodyweight, *ALBM* Albumine level, *Age_o18* Age over 18 years old, *Age_u18* Age under 18 years old, *BSA Body Surface Area*, CL_{CG} or CL_{CR} or *COCK* Creatinine clearance estimated by the original Cockcroft-Gault Equation, CL_{CRRT} Clearance of patients under CRRT (Continuous renal replacement therapy), CL_{CVVH} Clearance when patient is on CVVH (continuous venovenous haemofiltration), $CL_{EDD\text{-}f}$ Clearance when patient is on EDD-f (extended daily diafiltration) , *CLHDF* Clearance on hemodiafiltration, *CLHF* Clearance on hemofiltration, $CL_{NEDD\text{-}f}$ Clearance when patient is not on EDD-f, CL_{noCVVH} Clearance when patient is off CVVH, $CL_{n\text{-}CRRT}$ Clearance of patients not under CRRT, *CL_f* Clearance if patient is female, *CL_m* Clearance if patient is male, *FCR* Inverse of the final plasma creatine concentration recorded in μmol/L before commencement of EDD-f, *FFM* Fat-free mass, *HEIG* Height, *IBW* Ideal Bodyweight, *Q* Intercompartment clearance, *TBCL* Total Body Clearance, *TBW* Total Bodyweight, *V1_f* Apparent Volume of distribution of the central compartment if patient is female, *V1_m* Apparent Volume of distribution of the central compartment if patient is male, V_1 *or Vc* central volume, V_{CRRT} distribution volume of patients under CRRT, $Vn_{\text{-}CRRT}$ distribution volume of patients not under CRRT, *Vp* peripheral volume

[a] Of note, the author set the peripheral volume V_2 equal to V_1.

[b] Coefficient of variation, calculated as the square root of $(e^{\omega}-1)$*100%

[c] Variance of the residual error

[d] Estimated as ml/min

[e] Proportionality constant relating creatinine clearance (ml/min) to drug clearance (L/h)

[*] L/h/70 kg

Table S4. Covariates that were included or evaluated for inclusion by the population pharmacokinetic models included in this review.

Drug	Study	Tested and significant covariates																													
		Age	Sex	Height	Race	AdjBW[a]	AdjBW[b]	AdjBW[c]	TBW	IBW	BW$_{AD-t0}$	LBW	LBMc	FFM	BSA	BMI	SCr	hemoglobin	Salb	CL$_{CG}$	CL$_{hf}$	CL$_{hdf}$	CL$_{CKD-EPI}$	CL$_{MDRD}$	CalcCL$_{CR}$	CL$_{Robert}$	ARC	FCR	GFR	GFR$_{MDRD}$	GFR$_{CKD-EPI}$
Amikacin	Boidin C [16]	○	○	○			○	○	○	○					●	○	○			●										○	○
	Roger C [17]	○							● [d]		○						○		○		●	●									
	Carrié C [18]	○	○			●			○						○					●											
	Aréchiga-Alvarado NA [19]	○	○				○		○	●						○	○		○	●			○	○							
	Petitcollin A [20]	○	○						○								○			●											
	French MA [i] [21]																														
Gentamicin	Hodiamont CJ [22]	○	○	○						●							○		○	○					○						
	Teigen MM [23]								○	○										● [f]											
	Rea RS [24]	○	○		○				●								○												●		
	Bos JC [25]	○	○	○					○							○	○	○	○	●							○				
	Hodiamont CJ [26]							○	○	○																					
	Roberts JA [27]	○							● [g]			● [h]								○								●			
	Barletta JF [28]	○															○			○											
	Gomes A [29]	○	○	○					●			○	●	○	○					○											
	Watling SM [30]																														
	Kisor DF [i] [31]																														
	French MA [i] [21]																														
Tobramycin	Conil JM [32]			●						○							○			○						○					
	Aarons L [33]								○											●											
	Hennig S [34]	●	○	○					○					●			●			○											

ADJ Adjusted body weight, ALAT Alanine amino transferase, APACHE II Acute Physiology and chronic health evaluation II, *ARC* Augmented renal clearance (ARC) defined as a CLCR ≥ 130 mL/min, *ASAT* Aspartate amino transferase, *BMI* Body Mass Index, *BSA* Body Surface Area, *BUN* Blood Urea Nitrogen, *BW$_{AD-t0}$* Difference in patient's weight between the time of admission and the sampling day, *CalcCLCR* Creatinine clearance calculated from the creatinine concentration in a 6-h urine portion,*CBW* Corrected body weight, *CF* Cystic Fibrosis, *CLCKD-EPI* Creatinine clearance estimated with CKD-EPI, *CLCG* Creatinine Clearance estimated by Cockcroft-Gault equation, *CL$_{hdf}$* total amikacin clearance on hemodiafiltration, *CL$_{hf}$* total amikacin clearance on hemofiltration, *CL$_{MDRD}$* Creatinine clearance calculated with MDRD, *CL$_{Robert}$* Creatinine Clearance estimated by Robert equation, *CVVH* Continuous venovenous haemofiltration, *EDD-f* Extended daily diafiltration, *FCR* Inverse of the final plasma creatine concentration recorded in µmol/L before commencement of EDD-f, *FFM* Fat free mass, *Fluid$_{NPT}$* Amount of fluids collected by the NPT over the sampling day, *GFR* Glomerual filtration rate, *GFR$_{MDRD}$* GFR estmated by the equation fro the Modification of Diet in Renal Disease, GFR$_{CKD-EPI}$ GFR estimated by the equation from the Chronic Kidney Disease, *IBW* Ideal body weight, *ICU* Intensive Care Unit, *LBW* Lean body weight, *LBMc* Lean body mass according to the equation of Chennavasin (source), *NPT* Negative Pressure Therapy, *NSAIDs* Nonsteroidal anti-inflammatory drugs, SAlb Serum albumin, *SAPSII* Simplified acute physiology score II,*SCR* Serum Creatinine, *SOFA* Sepsis-related organ failure assessment score

● Tested and significant

○ Tested and not significant

[a] Adjusted body weight (ABW) was determined as follows : i) for BMI ≤ 30 kg/m^2 , ABW = TBW; ii) for BMI > 30 kg/m^2, ABW= ideal body weight (IBW) + 0.43 (TBW - IBW), with IBW calculated according to the Lorenz formula [74]

[b] Adjusted body weight was calculated as proposed by Bauer et al.: AdjBW = 0.4(TBW-IBW) +IBW for morbidly obese patients (IBW/TBW ratio of ≥ 1.9)

[c] Adjusted body weight proposed by Traynor et al. Was adapted according to French recommendations with a weight correction factor for overweight patients (IBW/TBW ratio of ≥ 1.25) and calculated as CBW = 0.43 (TBW-IBW)+IBW

[d] Described as Actual body weight

[e] Modified SOFA score (without neurologic and renal components)

[f] Creatinine Clearance estimated with Cockcroft-Gault with ideal body weight

[g] Normalized to 70 kg

[h] Normalized to 55 kg

[i] Covariate models were not used in this study

Table S4. Covariates that were included or evaluated for inclusion by the population pharmacokinetic models included in this review (continued).

Drug	Study	Tested and significant covariates																		
		SOFA score	SAPSII score	APACHE II score	ALAT	ASAT	Urea	BUN	Total proteins	Total bilirubin	Total daily diuresis	Serum electrolytes (sodium, potassium, chloride, calcium, phosphorus and magnesium)	24 hr fluid balance	Fluid balance since ICU admittance	Administration of total parental nutrition	Flow rate of ultrafiltrate during CVVH	Residual Renal function	Filter age	$Fluid_{NPT}$	Hemodialysis
Amikacin	Boidin C [16]	○	○		○	○														
	Roger C [17]																○	○		
	Carrié C [18]	○ [e]											○						○	
	Aréchiga-Alvarado NA [19]			○			○	○	○	○		○								
	Petitcollin A [20]																			
	French MA [i] [21]																			
Gentamicin	Hodiamont CJ [22]			○							○		○	○	○	○				
	Teigen MM [23]																			●
	Rea RS [24]																			
	Bos JC [25]																			
	Hodiamont CJ [26]																			
	Roberts JA [27]																			
	Barletta JF [28]																			
	Gomes A [29]																			
	Watling SM [30]																			
	Kisor DF [i] [31]																			
	French MA [i] [21]																			
Tobramycin	Conil JM [32]							○												
	Aarons L [33]																			
	Hennig S [34]																			

ADJ Adjusted body weight, ALAT Alanine amino transferase, APACHE II Acute Physiology and chronic health evaluation II, *ARC* Augmented renal clearance (ARC) defined as a CLCR ≥ 130 mL/min, *ASAT* Aspartate amino transferase, *BMI* Body Mass Index, *BSA* Body Surface Area, *BUN* Blood Urea Nitrogen, BW_{AD-t0} Difference in patient's weight between the time of admission and the sampling day, *CalcCLCR* Creatinine clearance calculated from the creatinine concentration in a 6-h urine portion,*CBW* Corrected body weight, *CF* Cystic Fibrosis, *CLCKD-EPI* Creatinine clearance estimated with CKD-EPI, *CLCG* Creatinine Clearance estimated by Cockcroft-Gault equation, CL_{hdf} total amikacin clearance on hemodiafiltration, CL_{hf} total amikacin clearance on hemofiltration, CL_{MDRD} Creatinine clearance calculated with MDRD, CL_{Robert} Creatinine Clearance estimated by Robert equation, *CVVH* Continuous venovenous haemofiltration, *EDD-f* Extended daily diafiltration, *FCR* Inverse of the final plasma creatine concentration recorded in µmol/L before commencement of EDD-f, *FFM* Fat free mass, $Fluid_{NPT}$ Amount of fluids collected by the NPT over the sampling day, *GFR* Glomerual filtration rate, GFR_{MDRD} GFR estmated by the equation fro the Modification of Diet in Renal Disease, $GFR_{CKD-EPI}$ GFR estimated by the equation from the Chronic Kidney Disease, *IBW* Ideal body weight, *ICU* Intensive Care Unit, *LBW* Lean body weight, *LBMc* Lean body mass according to the equation of Chennavasin (source), *NPT* Negative Pressure Therapy, *NSAIDs* Nonsteroidal anti-inflammatory drugs, SAlb Serum albumin, *SAPSII* Simplified acute physiology score II,*SCR* Serum Creatinine, *SOFA* Sepsis-related organ failure assessment score

● Tested and significant

○ Tested and not significant

[a] Adjusted body weight (ABW) was determined as follows : i) for BMI ≤ 30 kg/m^2 , ABW = TBW; ii) for BMI > 30 kg/m^2, ABW= ideal body weight (IBW) + 0.43 (TBW - IBW), with IBW calculated according to the Lorenz formula [74]

[b] Adjusted body weight was calculated as proposed by Bauer et al.: AdjBW = 0.4(TBW-IBW) +IBW for morbidly obese patients (IBW/TBW ratio of ≥ 1.9)

[c] Adjusted body weight proposed by Traynor et al. Was adapted according to French recommendations with a weight correction factor for overweight patients (IBW/TBW ratio of ≥ 1.25) and calculated as CBW = 0.43 (TBW-IBW)+IBW

[d] Described as Actual body weight

[e] Modified SOFA score (without neurologic and renal components)

[f] Creatinine Clearance estimated with Cockcroft-Gault with ideal body weight

[g] Normalized to 70 kg

[h] Normalized to 55 kg

[i] Covariate models were not used in this study

Table S4. Covariates that were included or evaluated for inclusion by the population pharmacokinetic models included in this review (continued).

		Tested and significant covariates																
										Usage of concomitant medication								
Drug	Study	Childhood (<18 years)	Study Site	Reason for admission	Usage of CVVH	Usage of mechanical ventilation	Disease presence (CF or no-CF)	Diagnosis of diabetes mellitus and/or arterial hypertension	Gas humidification	Vasopressin	Aminoside used	NSAIDs	Opiod analgesics	Cephalosporins	Diuretics	Antimycotics	Inotropics agents	Corticosteroids
Amikacin	Boidin C [16]																	
	Roger C [17]									○								
	Carrié C [18]									○								
	Aréchiga-Alvarado NA [19]					○		○	○			○	○	○	○	○	○	○
	Petitcollin A [20]																	
	French MA [i] [21]																	
Gentamicin	Hodiamont CJ [22]				●													
	Teigen MM [23]																	
	Rea RS [24]																	
	Bos JC [25]																	
	Hodiamont CJ [26]																	
	Roberts JA [27]																	
	Barletta JF [28]										●							
	Gomes A [29]																	
	Watling SM [30]																	
	Kisor DF [i] [31]																	
	French MA [i] [21]																	
Tobramycin	Conil JM [32]	○	○	○														
	Aarons L [33]																	
	Hennig S [34]						○											

ADJ Adjusted body weight, ALAT Alanine amino transferase, APACHE II Acute Physiology and chronic health evaluation II, *ARC* Augmented renal clearance (ARC) defined as a CLCR ≥ 130 mL/min, *ASAT* Aspartate amino transferase, *BMI* Body Mass Index, *BSA* Body Surface Area, *BUN* Blood Urea Nitrogen, BW_{AD-t0} Difference in patient's weight between the time of admission and the sampling day, *CalcCLCR* Creatinine clearance calculated from the creatinine concentration in a 6-h urine portion,*CBW* Corrected body weight, *CF* Cystic Fibrosis, *CLCKD-EPI* Creatinine clearance estimated with CKD-EPI, *CLCG* Creatinine Clearance estimated by Cockcroft-Gault equation, CL_{hdf} total amikacin clearance on hemodiafiltration, CL_{hf} total amikacin clearance on hemofiltration, CL_{MDRD} Creatinine clearance calculated with MDRD, CL_{Robert} Creatinine Clearance estimated by Robert equation, *CVVH* Continuous venovenous haemofiltration, *EDD-f* Extended daily diafiltration, *FCR* Inverse of the final plasma creatine concentration recorded in μmol/L before commencement of EDD-f, *FFM* Fat free mass, $Fluid_{NPT}$ Amount of fluids collected by the NPT over the sampling day, *GFR* Glomerual filtration rate, GFR_{MDRD} GFR estmated by the equation fro the Modification of Diet in Renal Disease, $GFR_{CKD-EPI}$ GFR estimated by the equation from the Chronic Kidney Disease, *IBW* Ideal body weight, *ICU* Intensive Care Unit, *LBW* Lean body weight, *LBMc* Lean body mass according to the equation of Chennavasin (source), *NPT* Negative Pressure Therapy, *NSAIDs* Nonsteroidal anti-inflammatory drugs, SAlb Serum albumin, *SAPSII* Simplified acute physiology score II,*SCR* Serum Creatinine, *SOFA* Sepsis-related organ failure assessment score

● Tested and significant

○ Tested and not significant

[a] Adjusted body weight (ABW) was determined as follows : i) for BMI ≤ 30 kg/m², ABW = TBW; ii) for BMI > 30 kg/m², ABW= ideal body weight (IBW) + 0.43 (TBW - IBW), with IBW calculated according to the Lorenz formula [74]

[b] Adjusted body weight was calculated as proposed by Bauer et al.: AdjBW = 0.4(TBW-IBW) +IBW for morbidly obese patients (IBW/TBW ratio of ≥ 1.9)

[c] Adjusted body weight proposed by Traynor et al. Was adapted according to French recommendations with a weight correction factor for overweight patients (IBW/TBW ratio of ≥ 1.25) and calculated as CBW = 0.43 (TBW-IBW)+IBW

[d] Described as Actual body weight

[e] Modified SOFA score (without neurologic and renal components)

[f] Creatinine Clearance estimated with Cockcroft-Gault with ideal body weight

[g] Normalized to 70 kg

[h] Normalized to 55 kg

[i] Covariate models were not used in this study

Model validation

Before assessing that a Pop-PK model is accurate for a specific population of patients, several steps should be completed beforehand to evaluate the model.

Firstly, pharmacokinetic parameters from the model to be evaluated are to be used to predict the concentration at the same sampling times of the new dataset. Based on these predictions, in order to assess predictive performance, goodness-of-fit plots of the predicted concentration and observed concentration should be done. Secondly, as part of assessing predictive performance, different metrics should also be estimated based on prediction error (PE) [71]. The latter can be calculated from the following equation:

$$PE = \frac{C_{pred_i} - C_{obs_i}}{C_{obs_i}} \times 100\% \qquad (1)$$

where C_{pred} and C_{obs} are the *i*th predicted and observed concentrations, respectively. In order to quantify bias, commonly used metrics are median or mean PE ($MDPE_i$) value based on the following equation:

$$MDPE_i\ (\%) = median\ or\ mean\ (PE_{ij}, j = 1, \ldots, N_i) \qquad (2)$$

From the values obtained for MADPE and MDPE, 95% confidence intervals should also be obtained to have a better overview of their plausible ranges. The 95% confidence intervals for MDPE should contain zero to be considered unbiased, whereas MADPE should be <20% to be deemed precise. The evaluation of these metrics also aims to determine if the Pop-PK model tends to under- or over-predict aminoglycosides concentrations. Considering that the consequences from an under-estimation of aminoglycosides concentrations (i.e. negative value of MDPE) could lead to nephrotoxicity and ototoxicity, the need of carefully evaluating a Pop-PK model before its application and generalization in other populations is even more crucial. On the other hand, an over-estimation of concentration could lead to a failure of reaching therapeutic targets of C_{max}/MIC for aminoglycosides.

Secondly, advanced internal validation, also known as interpolation, relies on simulations or subsets of the original dataset used for model building. Bootstraps, visual predictive checks (VPCs) or normalized prediction distribution error (NPDE) analysis are a few common strategies that should be considered to establish the overall fit of the Pop-PK model.
